# Cutaneous Myoepithelioma: An Unusual Tumor in the Hand

**DOI:** 10.1155/2020/3747013

**Published:** 2020-06-14

**Authors:** Nelson Montalvo, Ligia Redrobán, David Galarza, Iván Ramírez

**Affiliations:** ^1^Facultad de Ciencias Médicas de la Salud y la Vida, Escuela de Medicina, Universidad Internacional del Ecuador. Servicio de Patología, Hospital Metropolitano, Av. Mariana de Jesús s/n y Nicolás Arteta, Quito, Ecuador; ^2^Servicio de Patología Hospital Metropolitano, Quito, Ecuador; ^3^Facultad de Ciencias Médicas de la Salud y la Vida, Escuela de Medicina, Docencia y Departamento de Investigación, Universidad Internacional del Ecuador, Quito, Ecuador

## Abstract

Cutaneous myoepithelioma (CM) is a rare tumor among the primary skin neoplasms. We present the case of a patient with a diagnosis of CM in the right hypothenar region. Histological study showed a proliferation of myoepithelial cells with a solid, reticular growth pattern in a chondromyxoid stroma. The tumor cells were positive for CK AE, S-100, EMA, and p63.

## 1. Introduction

Proliferation of myoepithelial cells can lead to the formation of benign or malignant neoplasms. Cutaneous myoepitheliomas are recently described rare tumors originating in the skin, usually in the dermis. They can appear at virtually any age, most commonly in the upper and lower limbs. CM diagnosis can be challenging due to its different morphological presentations. Similarly, CMs have different immunohistochemical reactivity profiles, thus correct diagnosis depends on an adequate analysis. In general, cutaneous myoepithelioma cells show immunoreactivity for epithelial and myoepithelial markers. They are usually positive for EMA (epithelial membrane antigen), S-100 protein, and low molecular weight keratins. Reactivity is variable for glial fibrillary acidic protein (GFAP) and p63. Certain neoplastic cells maintain reactivity for EMA but are negative for keratins, while others may lose reactivity to myoepithelial markers like SMA (smooth muscle actin), and in rare cases, some can be negative for S-100 but positive for SOX-10. A syncytial variant of CM has been described with a different immunophenotype from that of the classical variant. These lesions have a good prognosis when resection is complete and the margins are negative.

## 2. Case Report

**Figure 1:**
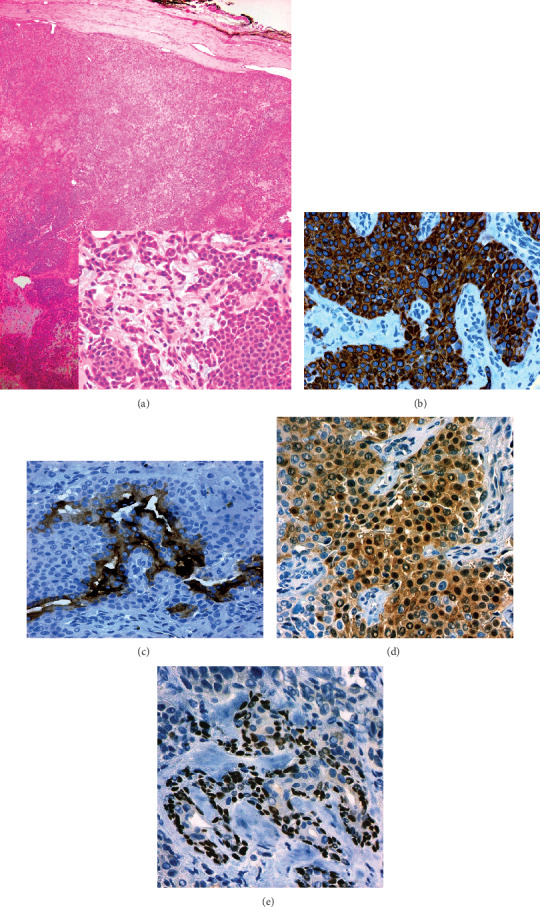
Cutaneous Myoepithelioma in the hypothenar region of the right hand. (a) A proliferation of myoepithelial cells with a solid and reticular growth pattern without cell atypia [insert HE 40X] in a chondromyxoid stroma. (b) The tumor cells show diffuse expression to cytokeratin AE1/AE3. (c) EMA focal expression. (d) Coexpression of S-100 protein. (e) p63 focal expression.

A 51-year-old male patient presented with a painless, progressively growing mass in the hypothenar region of the right hand with a one-year evolution. The lesion was completely resected. A pseudo capsulated dermal nodule of 1.5 × 1 × 0.4 cm with a grayish surface weighing 3 grams was received for pathological study. When cut, the lesion had a whitish, homogeneous appearance with hemorrhagic striae. Histological study (Figure 1) showed a proliferation of myoepithelial cells with epithelioid and plasmacytoid appearance, and a solid, reticular growth pattern in a chondromyxoid stroma. The tumor cells show diffuse expression to cytokeratin AE1/AE3, EMA focal expression, coexpression of S-100 protein and p63 focal expression. The mitotic activity was null. A peripheral connective tissue sheath around the lesion was also observed. The surgical margins showed no evidence of tumor cells.

## 3. Discussion

Myoepithelial cells are specialized basal cells that derive from the ectoderm. They are found in different tissues, including the sweat glands, salivary glands, mammary glands, and the respiratory tract [1]. In healthy skin, myoepithelial cells are arranged around the terminal portion of the secretory ducts of eccrine and apocrine sweat glands between the basement membrane and the secretory cells, forming a discontinuous cell layer that lines the outside of the glandular acini. These cells contract to help the extrusion of glandular content during the secretory phase [2–4]. Their morphology is variable and can present as spindle, epithelioid, plasmacytoid, or clear cells. Ultrastructurally, they show round or elongated nuclei with well-defined edges and central or eccentric localization. It is usual to observe desmosomes, pinocytic vesicles, and dense bodies formed by aggregates of cytoplasmic filaments. It is believed that these filaments play a determining role in contraction. It has been shown that myoepithelial cells produce fibronectin, laminin, and collagen, thereby contributing to maintenance of the surrounding extracellular matrix. Myoepithelial cells have the potential to differentiate into both myoid cells and epithelial cells. Therefore, their immunohistochemical reactivity is related to the lineage of differentiation. It is understood, then, that these cells show variable expression of vimentin, cytokeratins, epithelial membrane antigen (EMA), S-100 protein, muscle actins, and glial fibrillary acid protein (GFAP). It should be noted that myoepithelial cells do not usually express desmin [4]. Likewise, it is striking that some neoplastic myoepithelial cells, especially those with spindle cell morphology, sometimes show EMA positivity but cytokeratin negativity [3, 5, 6]. It has also been reported that as cells acquire neoplastic characteristics, they may lose their reactivity for muscle markers [7].

Myoepithelial neoplasms are a group of rare but well characterized tumors that includes, among others, salivary, skin, and soft tissue tumors [8]. The term *myoepithelioma* was coined for the first time in 1948 by Lever to describe a cutaneous neoplasm that would later be identified as a nodular hidradenoma, a lesion of entirely epithelial origin. Later on, a group of researchers showed that mixed tumors of the skin (or chondroid syringomas) contained a myoepithelial component represented by polygonal or plasmacytoid (hyaline) cells in a neoplasm with tubuloglandular differentiation. Given the variable cell morphology shown by the myoepithelial component of mixed tumors, researchers thought that the myoepithelial cells might be the origin of these neoplasms. Thus, the mesenchymal component of the mixed tumors was thought to originate from the myoepithelial cells lining the glandular epithelium of the lesions [9]. Recent evidence, however, suggests that this mesenchymal component has a mesodermal origin. Furthermore, the origin of the hyaline cells that are part of the mixed tumors has been attributed, at least in part, to myoepithelial lineage. Currently, cutaneous myoepitheliomas are recognized as neoplasms derived entirely from the myoepithelial cells without ductal differentiation [3]. Consequently, it would be plausible to think that cutaneous myoepitheliomas and chondroid syringomas with a prominent myoepithelial component represent two points along the same spectrum of cutaneous neoplasms [5].

Cutaneous myoepitheliomas occur more frequently in men, as is also the case with chondroid syringomas [3, 4, 10, 11]. However, while most of the latter occur on the head and neck, CMs are more frequently located on the extremities and have a wider anatomical distribution, including the scalp, head, neck, chest, and back [12–14]. A recent review indicated that CM appears not to have a sexual predominance. However, this fact seems to be related to all myoepitheliomas of the skin and soft tissues and not to CM as an individual entity. The average age of presentation is 22.5 years, although they may present at any age. Two peaks of incidence have been identified: one between the first and second decades of life and the other between the third and fifth. Only 20% of reported myoepithelial neoplasms occurred in pediatric patients, and half of these showed malignant behavior [15, 16].

Clinically, CM usually presents as a painless nodule of variable size and gradually progressing growth that generally causes no problems or discomfort. This description coincided with our patient's clinical presentation and with that described by Kanemaru et al. regarding a cutaneous myoepithelioma on the right arm of a Japanese male patient [17]. An abrupt increase in tumor size or a sudden change in growth rate should arouse suspicion of malignant behavior. It has been reported that a benign component can be found within the tumor lesion in 24% to 50% of myoepithelial carcinomas in the salivary glands, suggesting the possibility of malignant transformation. This theory has not been validated for cutaneous myoepitheliomas. Although the histological similarity between CM and cutaneous myoepithelial carcinoma (CMC) would support the identification of a morphologically benign area in a CMC, it is very difficult to do so because, unfortunately, no specific criteria have yet been established for such a differentiation, which is probably because of the rarity of these tumors. Using cellular atypia as an indicator of malignancy, it has been reported that less than 1% of CMC cases show a morphologically benign component in the main lesion. Further, considering the predominance of CM in the male sex, we would expect most CMCs to also occur in males if they in fact came from a preexisting benign lesion. This assumption, however, is not borne out, since CMCs appear more frequently in women, at a ratio of 2 : 1 [8, 13, 18].

Macroscopically, CM shows up as a well-defined, usually whitish-gray or yellowish lobular lesion with a smooth surface and no capsule. It varies in size, although it is usually smaller than CMC, and can range from 0.5 to 2.5 cm, averaging at 0.7 cm [6]. The consistency of the tumor may be firm, meaty, or gritty. Areas with calcifications may be present. Necrosis and hemorrhage are not common in benign neoplasms, even though cases of CM with areas of necrosis have been reported. This finding, however, was not related to recurrence or malignant behavior of the lesion.

Histologically, CMs appear as well-defined nodular, multinodular, or lobular neoplasms with no capsule that usually show infiltration. A minority of cases are exophytic lesions with an epidermal collarette. In the present case, a peripheral connective sheath was observed, even though the lesion was not exophytic. The superficial subcutaneous tissue is compromised in around 25% of cases [11]. It is typical of this type of tumor to show different structural growth patterns. Thus, a proliferation of fusiform, oval, or epithelial cells can be observed forming sheets, sheaths, cords, or bundles, in a myxoid, chondromyxoid, or hyaline stroma [19]. It is common to find foci with solid growth and individual infiltrating cells. Certain tumors may show a predominant growth pattern, the solid and spindle cell patterns being the most frequent. In our case, the solid and reticular patterns predominated. It is common to find different cellular patterns and morphologies within the same lesion. Swali et al. reported a CM occurring on the medial aspect of the right foot in a 39-year old Hispanic male. The tumor showed poorly defined architecture with cells arranged in fascicles and nodules [20]. To date, only one case with a plexiform pattern has been reported [10]. Other known morphological patterns include that of hyaline (plasmacytoid) cells, vacuolated cells with clear cytoplasm, and rhabdoid cells. Heterologous differentiation has been documented in up to 15% of cases. Chondroid metaplasia is the most frequent, in general. Adipose metaplasia seems to be more frequent in cases of the syncytial variant of CM [21]. The neoplastic cells usually show clear or eosinophilic cytoplasm with round or oval nuclei, mild or no cellular atypia, and small or indistinguishable nucleoli. Mitotic figures are rare and the mitotic rate is usually low (up to 4 mitoses per HPF).

In CMs, tumor cells show coexpression of epithelial markers and S-100 protein, as in our case. Most show positivity for cytokeratins (pan-keratin, AE1/AE3, Cam5.2), while positivity for EMA has been variably reported in from 19% to 79% of cases. S-100 protein is also positive in most cases (72%–100%). The reactivity for GFAP is lower, and can be positive in up to half of cases. Myogenic marker expression is variable. Calponin is positive in 86% to 100% of cases, while SMA is positive in only 36% to 64%. This type of tumor usually does not express desmin, although a few positive cases have been reported for this marker (0%–20%). P63 is positive in up to 70% of cases, and SOX-10 is present in 82%. This last marker is especially useful in cases that are negative for S-100 protein [5]. Interestingly, CMs of the syncytial variant have a particular immunophenotype that differs from the classical variant (See Table 1).

Rearrangements of the *EWSR1* gene, located on the long arm of chromosome 22, have been reported in 45% of all myoepitheliomas and myoepithelial carcinomas of the skin and soft tissues [6, 9, 13, 16, 22]. This percentage rises to 80% for the syncytial variant of CM. Several fusion genes have been identified for the classic CM variant; among them, *POU5F1*, *PBX1*, and *ATF1*. Although the biological importance of these gene fusions is not yet known for cutaneous myoepitheliomas, there seems to be a relationship between a specific gene fusion and tumor morphology in soft tissue myoepitheliomas. The finding of molecular alterations involving *EWSR1* in mixed cutaneous tumors, CMs, and some CMCs emphasizes the relatedness of these entities within a single spectrum [14].

In the context of a morphology suggestive of myoepithelial neoplasia, a CM diagnosis requires a complete immunohistochemical study. It is clear that these tumors are characterized by the wide range of histological patterns and immunophenotypes they can show, which sometimes complicates differential diagnosis with other skin neoplasms such as epithelioid fibrous histiocytoma, early juvenile xanthogranuloma (without lipidization), Spitz nevus, epithelioid sarcoma, and ossifying fibromyxoid tumor. Differential diagnosis will depend on the CM's predominant histological pattern (see Table 2). Briefly, epithelioid fibrous histiocytoma shows binucleated cells in a well-vascularized stroma present to a greater degree than in CM. Although it can be positive for EMA, it is negative for S-100 protein and shows reactivity for ALK. Early stage JXG lacks the multinucleated giant cells and lipidization that are often key to their identification. It is normal to find inflammatory cell infiltrates (lymphocytes and eosinophils) in the lesion, and this tumor is usually negative for EMA and S-100. The Spitz nevus is, as a rule, a cell proliferation forming nests that decrease in cellularity with depth. While it is positive for S-100, it is consistently negative for EMA and GFAP. It also shows reactivity for Melan-A. Epithelioid sarcoma ordinarily shows significant local infiltration towards fibrous septa and fascias. It is also typically positive for EMA and keratins, but negative for S-100 and GFAP. Ossifying fibromyxoid tumors generally present as lobular lesions of round or oval cells, frequently with a peripheral halo of bone metaplasia. It shows positivity for S-100 and half of cases are positive for desmin [11, 13].

The clinical course of myoepithelioma does not always correlate with their histological characteristics. Most metastatic skin and soft tissue myoepitheliomas showed cellular atypia, high mitotic rate, and infiltrating margins, but a small number of cases without these characteristics showed unusual malignant behavior [7, 11]. There appears to be a relationship between a high mitotic rate and the risk of recurrence or metastasis in tumors with benign cytology in CM cases [13]. Cell atypia is the only recognized criterion of malignancy and is also the best predictor of malignant behavior. In the absence of atypia, a high mitotic rate, dense chromatin, prominent nucleoli, nuclear pleomorphism, and necrosis denote a greater risk of malignancy [15, 23]. The reported CM recurrence rate is 20% and, in general, even after a recurrence, the clinical course is usually favorable. In view of the above, the treatment of choice is complete resection of the lesion with negative margins [16, 24].

## 4. Conclusion

We present this case to highlight the need to consider myoepithelioma within the tumors of hand. The correct histological diagnosis in a very rare location ensures adequate management and follows up.

## Figures and Tables

**Table 1:** Main characteristics of the classic and syncytial variants of cutaneous myoepithelioma∗^1^.

	Cutaneous myoepithelioma—classical variant	Cutaneous myoepithelioma—syncytial variant
Growth pattern	Trabecular, reticular, plexiform (rare)	In sheets, solid, and syncytial
Cytology	Mixed: epithelioid, fusiform, clear, or plasmocytoid cells	Ovoid, fusiform, or histiocytoid
Stroma	Chondromyxoid, myxoid, or hyaline	Sparse; presence of adipose metaplasia seems to be more frequent than in the classical variant
Immunohistochemistry		
Calponin	(+) 86%–100%	(+) >85%
SMA	(+) in up to 60% of cases	(+) 70%
Desmin	(-) 80%–100%	(-) 80%–100%
EMA	(+) 42%	(+) 100%
Cytokeratins (pankeratin, AE1/AE3, Cam5.2)	Diffuse and intense positivity in most cases (93%–100%)	Focal positivity in a few cases (12%)
S-100	(+) 72%–100%	(+) 86%–100%
GFAP	(+) variable, 27%–54%	(+) 42%
p63	(+) variable, 7%–45%	(+) 54%
Molecular alteration	EWSR1 gene rearrangement. Identified fusion genes include PBX1, PBX3, POU5F1, ZNF444, DUX4, ATF1, NR4A3, CREB1.	EWSR1 gene rearrangement. The fusion genes are different from the classical variant.

∗^1^Table references: 5 ,9 ,12 ,13 ,14.

**Table 2:** Differential diagnosis of cutaneous myoepithelioma∗^2^.

Tumor	Age of presentation	Most frequent location	Form of the lesion	Histology	Cytology	Immunohistochemistry	Molecular alteration
For CM with sheets of epithelioid, ovoid, or histiocytoid cells and solid growth pattern							
Benign epithelioid fibrous Histiocytoma	Adults, second to fifth decades	Lower limbs and trunk	Exophytic, polypoid	Highly vascularized stroma with scattered inflammatory cells. Trapping of collagen bundles at the periphery of the lesion.	Epithelioid cells, often binucleated	S-100 (-), keratin (-), EMA (+/-), SMA (-/+), calponin (-)	AKL gene rearrangement. Identified fusion genes include SQSTM1, VCL, DCTN1, ETV6, PPFIBP1 and SPECC1L
Spitz nevus	Frequent in children; 65% of all cases occur in adolescents and young adults	Head and neck, trunk and lower limbs	Semispherical nodule	Solid pattern with cellular nests. Kamino bodies.	Epithelioid melanocytes with prominent nucleoli	S-100 (+), HMB45 (+), Melan-A (+), keratin (-), EMA (-), GFAP (-)	Alteration of the HRAS gene has been reported
Epithelioid sarcoma	Young adults	Distal portion of the extremities	Exophytic	Multinodular, very evident and diffuse infiltration with no myxoid or hyalinized stroma	Epithelioid to fusiform	Keratin (+), EMA (+), S-100 (-), GFAP (-)	Deletions of SMARCB1; gain of chromosomes 11, 1, 6, and 9 have been reported

For CM composed predominantly of spindle cells							
Pilar leiomyoma	Young adults; may be congenital	Limbs and trunk	Nodule (usually painful)	Cellular bundles ramifying between collagen fiber bundles	Fusiform	Desmin (+), S-100 (-), GFAP (-), keratin (+/-), EMA (+/-)	Not reported

For CM showing reticular pattern and myxoid matrix							
Ossifying fibromyxoid tumor	Adults, fifth decade of life	Lower and upper limbs	Exophytic	Lobular o multinodular	Round or ovoid	S-100 (+), desmin (+), keratins (+/-), GFAP (+/-)	PHF1 gene rearrangement
Extraskeletal myxoid chondrosarcoma	Adults, peak in the sixth decade	Limbs, especially the groin and gluteal region	Exophytic	Lobular or multinodular; reticular or trabecular pattern. Greater cellularity towards the periphery of the nodules.	Fusiform to epithelioid	Keratins (-), p63 (-), GFAP (-), SMA (-), EMA (-/+), S-100 (-/+)	Translocation t (9; 22) (q22; q12)–NR4A3-EWSR1.

∗^2^Table references: 5, 9, 12, 18, 19.
